# Supplementary material for: scGRNom: a computational pipeline of integrative multi-omics analyses for predicting cell-type disease genes and regulatory networks
Source: Genome Med. 2021 May 27;13:95. doi: 10.1186/s13073-021-00908-9 (PMC8161957; doi:10.1186/s13073-021-00908-9)
Supplement: Supplementary file 3 — Additional file 3. Supplementary figures (Figures S1-S3) and tables (Tables S1-S2). [file 13073_2021_908_MOESM3_ESM.docx]

**Fig. S1 – Comparison with PIDC, GENIE3, and GRNBoost2.** Given a cell-type GRN by scGRNom, we selected top K TFs per target gene (TG) to see if the TF-TG pairs were also predicted by PIDC, GENIE3, or GRNBoost2 (also picking top K TFs per TG). We then calculated the percentages of the scGRNom’s TF-TG pairs that can be predicted by one of those methods. Actually, those methods typically generated more network edges than us because scGRNom only keeps the TF-TG links in which TFs have binding sites on the regulatory elements (e.g., enhancers and promoters). To make those networks comparable with scGRNom, we varied K values from 0% up to 30% (x-axis). The y-axis showed the percentages of the scGRNom’s TF-TG pairs can be predicted by PIDC, GENIE3, or GRNBoost2. Each curve represents a cell type. (**A**) scGRNom’s cell-type GRNs without considering open chromatin regions from scATAC-seq. (B) scGRNom’s cell-type GRNs only including the edges with the enhancers overlapping cell-type open chromatin regions from scATAC-seq.

**Fig. S2 – Partitioned heritability enrichments of GWAS SNPs associated with various diseases and traits (bar) on the enhancers of cell-type GRNs.** Bar height is -log10(*p* value) of the enrichment. The diseases and traits are Schizophrenia (SCZ), Alzheimer’s disease (AD), Autism spectrum disorder (ASD), Bipolar disorder (BPD), Amyotrophic lateral sclerosis (ALS), Major depressive disorder (MDD), Intelligence, Multiple sclerosis (MS), Parkinson’s disease (PD), Attention deficit hyperactivity disorder (ADHD), Education, Type 2 diabetes (T2D), Inflammatory bowel disease (IBD), Coronary artery disease (CAD). The red line represents *p* value = 0.05.

**Fig. S3 – Numbers of cell-type disease genes shared by numbers of cell types.** The x-axis represents the number of cell types for counting shared cell-type disease genes. Bar height is number of shared disease genes (y-axis). The diseases and Top: Schizophrenia (SCZ). Bottom: Alzheimer’s disease (AD)

**Table S1:** The numbers of GWAS SNPs that interrupt the binding sites of at least one of all possible TFBSs and the binding sites of the regulatory TFs in each cell-type GRN.

| Cell type | AD SNPs interrupting at least one of all possible TFBSs | AD SNPs interrupting regulatory TFBSs of cell type GRN | SCZ SNPs interrupting all possible TFBSs | SCZ SNPs interrupting regulatory TFBSs of cell type GRN |
| --- | --- | --- | --- | --- |
| Microglia | 455 | 81 | 1102 | 110 |
| Oligodendrocyte | 276 | 24 | 1381 | 329 |
| Ex1 | 204 | 35 | 2120 | 414 |
| Ex2 | 204 | 23 | 2140 | 320 |
| Ex3e | 204 | 21 | 2115 | 253 |
| Ex4 | 204 | 23 | 2131 | 414 |
| Ex5b | 204 | 32 | 2140 | 310 |
| Ex6a | 199 | 7 | 1874 | 222 |
| Ex6b | 203 | 9 | 2122 | 249 |
| Ex8 | 201 | 7 | 1972 | 336 |
| Ex9 | 196 | 24 | 1887 | 270 |
| In1a | 165 | 0 | 1755 | 131 |
| In1b | 201 | 32 | 2038 | 242 |
| In1c | 203 | 18 | 2077 | 321 |
| In3 | 204 | 13 | 2101 | 337 |
| In4a | 196 | 12 | 1983 | 165 |
| In4b | 200 | 19 | 2072 | 412 |
| In6a | 196 | 6 | 1938 | 180 |
| In6b | 204 | 15 | 2125 | 405 |
| In7 | 199 | 25 | 1978 | 238 |
| In8 | 204 | 26 | 2115 | 253 |

**Table S2: Hypergeometric test for the overlap significance between scGRNom and public gene regulatory networks (GRNs)**. The public GRN databases include TRRUST [59], Dorothea [60] and RegNetwork [61]. We used the hypergeometric test to calculate *p*-values quantifying the significances of the overlaps (TF-TG edges) between the scGRNom’s network and those public general GRNs. In particular, we assume that the scGRNom network has $S$ edges, a public GRN has $M$ edges, and they overlap $K$ edges. Also, there are $N$ possible TF-TG edges in total. Then the *p*-value of the overlap significance by hypergeometric test is given by $p\left( K,S,M,N \right)=\sum_{i=K+1}^{M} \frac{\left( \begin{matrix} S \\ i \end{matrix} \right)\left( \begin{matrix} N-S \\ M-i \end{matrix} \right)}{\left( \begin{matrix} N \\ M \end{matrix} \right)}$. Here, $S$ = 242535 for all cell-type networks by scGRNom. We use the minimum value of $N=M+S-K$ and the larger $N$ values even increase the *p*-value.

| Public GRN | Number of edges ($M$) | Number of overlapped edges with scGRNom ($K$) | Number of total edges ($N$) | Hypergeometric *p*-value |
| --- | --- | --- | --- | --- |
| TRRUST | 9396 | 319 | 251612 | >0.999 |
| Dorothea | 489552 | 5935 | 726152 | >0.999 |
| RegNetwork | 3954 | 106 | 246383 | >0.999 |
